# Two new species of *Prolyda* from the Middle Jurassic of China (Hymenoptera, Pamphilioidea)

**DOI:** 10.3897/zookeys.569.7249

**Published:** 2016-02-26

**Authors:** Chen Wang, Chungkun Shih, Alexandr P. Rasnitsyn, Mei Wang

**Affiliations:** 1 College of Life Sciences, Capital Normal University, 105 Xisanhuanbeilu, Haidian District, Beijing 100048, China; 2 Palaeontological Institute, Russian Academy of Sciences, 123, Profsoyuznayaul, Moscow 117997, Russia; 3 Department of Palaeontology, Natural History Museum, Cromwell Road, London SW7 5BD, UK

**Keywords:** Daohugou, fossil insect, Jiulongshan Formation, sawfly, taxonomy

## Abstract

Two new species of the genus *Prolyda* Rasnitsyn, 1968, *Prolyda dimidia* **sp. n.** and *Prolyda elegantula* **sp. n.**, are described and illustrated. Both specimens were well-preserved and collected from the latest Middle Jurassic Jiulongshan Formation of Daohugou Village in Inner Mongolia, China. Based on the new morphological data, a key to the five known species of *Prolyda* is provided. In addition, *Prolyda* has an enlarged first antennal flagellomere, which means it might have revert to the elongate plesiomorphic state for the antennal configuration as previously documented.

## Introduction

Xyelydidae have long been regarded as the basal group of the Pamphilioidea (Rasnitsyn 1980, 1983, 2002; Grimaldi and Engel 2005). However, Wang et al. (2015a) conducted a phylogenetic study on Pamphilioidea with 33 extinct and 11 extant genera based on 45 morphological characters. The results did not support Xyelydidae as a monophyletic group – *Xyelyda* was shown to be a sister taxon to the other pamphilioids, while the relationship among the remaining xyelydids was mostly unresolved, except for the three genera of *Strophandria*, *Prolyda* and *Medilyda*, which, together with Pamphiliidae, formed a monophyletic clade defined by the synapomorphy of having 1r-m of hind wing in line with 1-M (Wang et al. 2015a, fig. 3). Because Xyelydidae is a paraphyletic group as a family and *Strophandria*, *Prolyda* and *Medilyda* form a monophyletic clade with Pamphiliidae, *Prolyda* is tentatively referred to Pamphilioidea *incertae sedis*, pending a formal reclassification of the superfamily.

A total of 12 genera and 30 species of xyelydids has been reported to date (table 1 in Wang et al. 2015a), most of which are distributed in Kyrgyzstan, P. R. China, Kazakhstan, and Russia. Most xyelydids have been reported from the Jurassic, with the oldest representatives *Sagulyda* spp. and *Ferganolyda* spp. from the Lower or Middle Jurassic Sogul Formation in Kyrgyzstan (Rasnitsyn 1983, Rasnitsyn et al. 2006, Wang et al. 2015b). Only three genera (*Novalyda*, *Fissilyda* and *Rectilyda*) of xyelydid have been described in the Early Cretaceous (Gao et al. 2013, Wang et al. 2015c); these are the latest occurrences of xyelydids in the fossil records.

The Yanliao biota at the Daohugou site has become well known because of the recent discoveries and reports from this locality a variety of excellently-preserved insects, plants, and other animals (Ren et al. 2010, 2012), among which Hymenopterans are especially well represented (Gao et al. 2009, Shih et al. 2009, Wang et al. 2012, Li et al. 2014). The age of these fossil-bearing beds is considered to belong to the latest Middle Jurassic (Bathonian-Callovian boundary), about 165 – 164 million years before present (Walker et al. 2013). We herein describe two new species of *Prolyda* Rasnitsyn, 1968, *Prolyda dimidia* sp. n. and *Prolyda elegantula* sp. n., based on two new specimens from the Daohugou beds, which expand the previously known geographical distribution of Prolyda and move their existence period further back in time.

## Materials and methods

Both type specimens are deposited in the Key Laboratory of Insect Evolution and Environmental Changes, College of Life Sciences, Capital Normal University, Beijing, China (CNUB; Dong Ren, Curator).

The specimens were examined and photographed, either dry or moistened with 95% ethanol, with a Leica DFC500 digital camera attached to a Leica MZ165C dissecting microscope (Leica, Wetzlar, Germany). The wing venation nomenclature used in this study is modified from Rasnitsyn (1969, 1980). Venation symbols: SC, R, RS, RS+M, M, Cu, M+Cu are main (longitudinal) veins; 1-RS, 1-M *etc.* are sections of these veins; 1r-rs, 2r-rs, 2r-m, *etc.* are cross veins; 1r, 2rm, 1mcu, *etc.* are cells.

## Taxonomy

### Hymenoptera Linnaeus, 1758
Pamphilioidea Cameron, 1890 Family *incertae sedis*

#### Prolyda

Taxon classificationAnimaliaHymenopteraXyelydidae

Rasnitsyn, 1968

##### Type species.

*Prolyda karatavica* Rasnitsyn, 1968

##### Other species included.

*Prolyda depressa* Rasnitsyn, 1969, *Prolyda xyelocera* Rasnitsyn, 1968, *Prolyda dimidia* sp. n., and *Prolyda elegantula* sp. n.

##### Amended diagnosis.

Head massive, circular or cube-like; mandibles curved, strong and sickle-like; pronotum short and wide; the first antennal flagellomere equal to head in length, but eight times as long as the second flagellomere; forewing pterostigma variable, completely sclerotized or partly sclerotized, or just membranous; M diverging from M+Cu at much larger angle than Cu; 1-RS proclival or somewhat vertical; angle between 1-M and RS+M almost 90°; 1cu-a distal to the middle of cell 1mcu or located at middle; 2r-rs almost in line with 2r-m; hind wing with 1r-m rather long, as long as or slightly shorter than 1-M.

#### Prolyda dimidia sp. n.

Taxon classificationAnimaliaHymenopteraXyelydidae

http://zoobank.org/EF0FFA89-C470-469F-ACAF-371106977ED3

Figs 1 , 2

##### Diagnosis.

In addition to generic diagnosis, SC1 longer than SC2; SC2 relatively long, almost equal to 1-RS in length; 1-M short, about twice as long as 1-RS, and 0.6 times as long as RS+M; 2r-m slightly postfurcal; 1r-rs vertical, slightly shorter than 2r-rs and parallel to it; 3r-m located well distal to middle of cell 3r, separated from apex of cell 3r by almost its own length; 2m-cu at middle of cell 3rm.

##### Measurements

**(in mm).** Body length (excluding antenna) 11.4, head length including mandible 2.03, width 2.48, forewing length up to the end of cell 3r 8.92, hind wing length up to the end of cell r 6.58.

##### Description.

**Figure 1.**
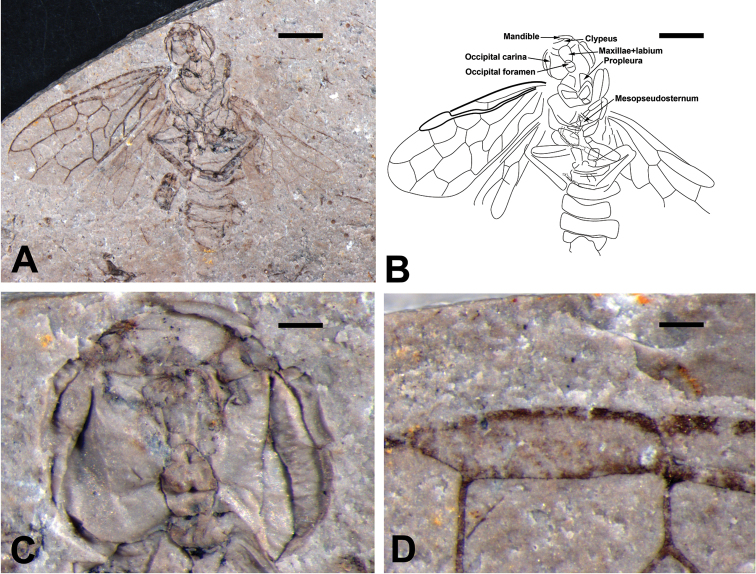
*Prolyda dimidia* sp. n. Holotype: **A** Photo of habitus **B** Line drawing **C** Head **D** Pterostigma. Scale bars: 2 mm (**A, B**); 0.2 mm (**C, D**).

Color not reliably known because of absence of counterpart (Fig. 1A). As preserved, body and legs moderately pale except for tibiae and tarsi dark; forewing somewhat infuscated subbasally, particularly so in costal area along veins, pterostigma slightly infuscate.

Head circular and large (Fig. 1C), and nearly 1.38 times as wide as mesothorax; clypeus distal margin straight; mandibles incompletely preserved. Propleura large and rectangular, structure of meso- and metathorax unknown except for mesopseudosternum triangular, far from anterior margin of ventropleuron.

Three pairs of legs preserved (Fig. 1B); coxae trapezoid, and hind coxae elongate; femora short and fusiform, fore femur slightly thicker than tibia, approximately 3 times as long as wide, covered with dense bristles; tibia short and narrow, mid tibia about 1.4 times as long as mid femur; tarsi incompletely preserved, fore tarsi with 1^st^, 2^nd^ and 5^th^ segment elongate, 3^rd^ and 4^th^ subtriangular, with length of fore tarsal segments 1:2:3:4:5 = 3.98: 1.57: 1.18: 1: 1.46, hind tarsal segments 1: 2: 3: 4 = 4.16: 1.69: 1.35: 1.

**Figure 2.**
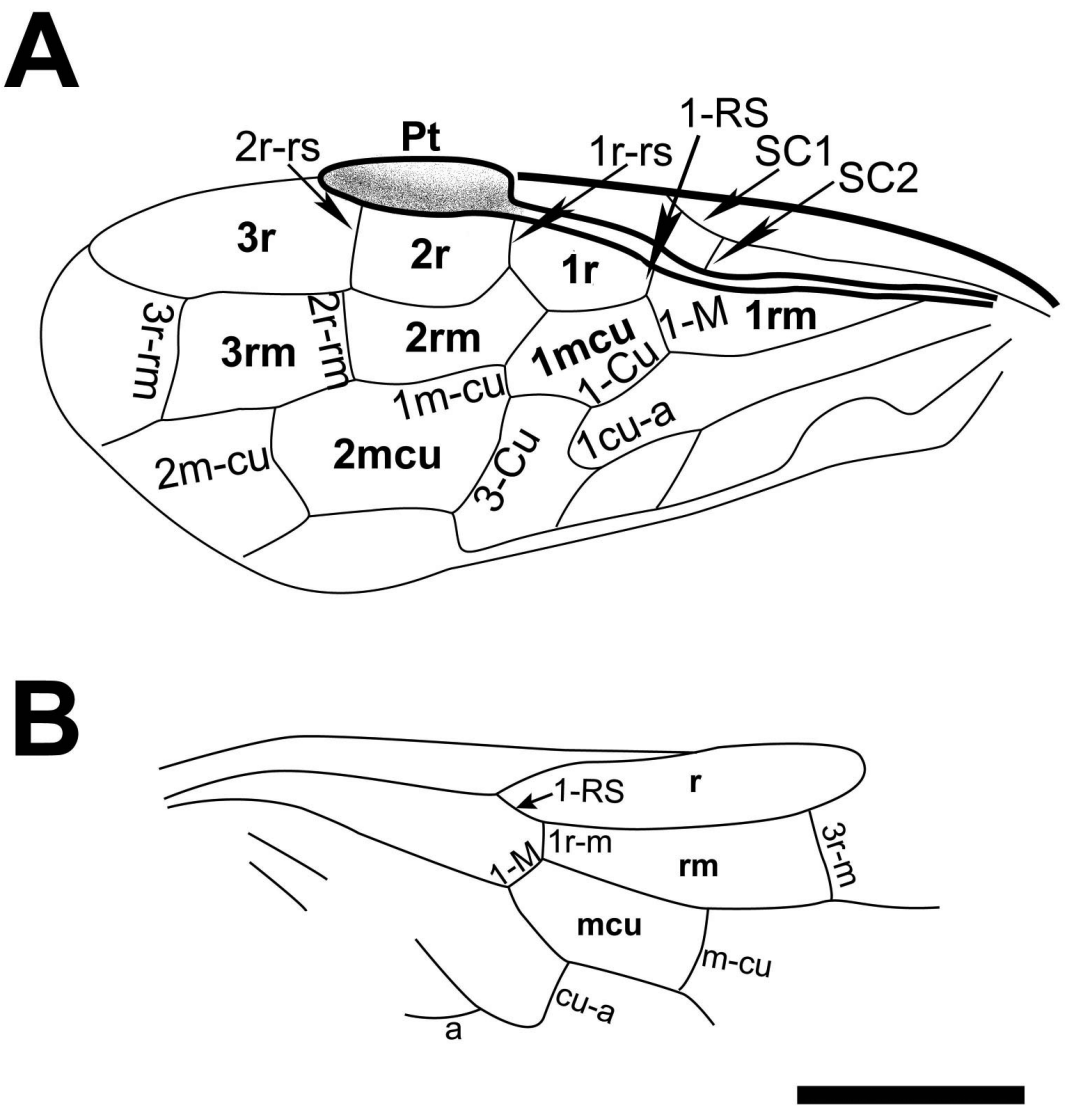
Interpretation of the wings of *Prolyda dimidia* sp. n. Holotype: **A** Left forewing **B** Right hind wing. Scale bar: 2 mm.

Forewing (Fig. 2A) with pterostigma sclerotized around margins (Fig. 1D), posterior part thicker than anterior; SC with two branches, anterior branch merging with C at origin of 1-RS, posterior branch long and oblique, slightly longer than 1-RS; R curved proximal to RS base; 1-RS almost vertical, about half of 1-M; cross vein 1r-rs vertical, 0.6 times as long as 2r-rs and parallel to it; 2r-m almost interstitial, located distal to middle of cell 2mcu; 3r-m separated from the apex of cell 3r by almost its own length; M+Cu curved; RS+M vertical to 1-M, 1.8 times as long as 1-M, and nearly of same as 1-Cu; 2-Cu curved upwards, almost as long as 2-M; 1m-cu 0.43 times and 0.25 times as long as 2-Cu and 3-Cu, respectively; 1cu-a bent distinctly towards wing apex, equal to length of 2-Cu, slightly distal to middle of cell 1mcu; 2m-cu curved nearly at its mid length, located at middle of cell 3rm; cell 1mcu 1.8 times as long as wide, 0.6 times as long as cell 2rm; cell 2rm nearly as long as cell 3rm, and cell 3r 1.7 times as long as cell 2r.

Hind wing (Fig. 2B) SC absent; cell r tapering apically; 1-RS about as long as 1-M; cross vein 1r-m far from bases of both RS and M; 3r-m oblique towards wing apex, separated from apex of cell r by 0.96 times of its own length; cross vein m-cu equal to length of 3r-m, located distal to middle of cell rm; cross vein cu-a nearly at middle of cell mcu; vein M+Cu almost straight and 1A arched upward, cross vein a distant from cu-a.

##### Material examined.

**Holotype**, No. CNU-HYM-NN-2012147.

##### Distribution.

Jiulongshan Formation; Daohugou Village, Shantou Township, Ningcheng County, Inner Mongolia, China (41°18.979’N, 119°14.318’E); latest Middle Jurassic of the Bathonian-Callovian boundary.

##### Etymology.

The species epithet is derived from the Latin word “dimidius”, meaning half, referring to the pterostigma being more infuscate in the posterior half.

#### Prolyda elegantula sp. n.

Taxon classificationAnimaliaHymenopteraXyelydidae

http://zoobank.org/4CF3DA7F-BADE-4055-B3EB-86C69CD507B2

Fig. 3

##### Diagnosis.

In addition to generic diagnosis, SC1 almost as long as SC2; 1-M long, about 0.8 times as long as RS+M; 2r-m well postfurcal; 1r-rs proclival and half as long as 2r-rs; 3r-m near apex of cell 3r, separated from apex of cell 3r by half of its length; cell 3rm widening towards apex; 2m-cu distal to middle of cell 3rm.

##### Measurements

**(in mm).** Body length (excluding antenna) 12, head length including mandible 2.36, width 2.88, forewing length up to the end of cell 3r 7.02, hind wing length up to the end of cell r 4.8.

##### Description.

**Figure 3.**
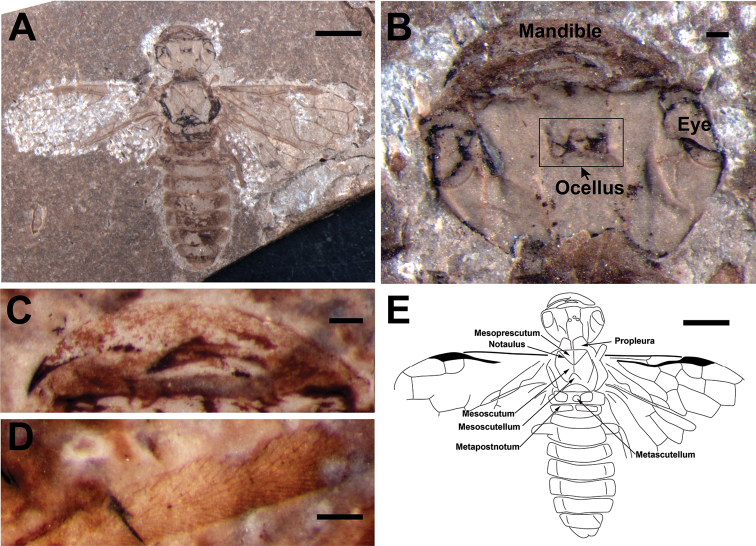
*Prolyda elegantula* sp. n. Holotype: **A** Photo of habitus **B** Head **C** Mandible **D** Setae on the hind tibia **E** Line drawing. Scale bars: 2 mm (**A, E**); 0.2 mm (**B–D**).

Color not reliably known because of absence of counterpart (Fig. 3A). As preserved, body infuscated with part of head, mesonotum and abdomen paler than pterostigma.

Head massive and wide (Fig. 3B), about 1.5 times as wide as mesonotum, 1.73 times as wide as long (excluding mandibles); eyes oval, extending forward to mandible base, 1.6 times as long as wide, with three ocelli forming obtuse triangle; anterior clypeal margin slightly wavy; mandible (Fig. 3C) sickle-shaped, bent, almost reaching opposite side of head when closed, with long, strong apical tooth and short subapical one, almost at midlength of mandible.

Prothorax with propleura large and rectangular; as wide as mesoprescutum; mesonotum with median mesoscutellar sulcus and notauli present, mesoprescutum large, 0.4 times as long as mesonotum and 1.5 times as long as mesoscutellum; metanotum with metascutellum almost circular and metapostnotum rectangular, about 3.3 times as long as wide. Femora wide, fusiform and strong; hind femur about twice as wide as fore femur, and twice as wide as hind tibia; tibia covered with thick bristles (Fig. 3D).

Abdomen without tergum split, wider than mesonotum; abdominal segments narrow, and parallel-sided; anterior margin of the first segment incurved. Genitalia not preserved.

Forewing (Fig. 3E) pterostigma completely sclerotized; SC located at middle of cell C, with two branches, anterior and posterior branches of almost equal length, posterior one oblique, parallel to 1-RS and of same length; R distinctly angular at RS base; 1-RS proclival, about 0.45 times as long as 1-M; 1-M subvertical to RS+M, and 0.83 times as long as RS+M ; 1r-rs short and oblique, half as long as 2r-rs, with RS arching posteriorly between them; M+Cu curved; 1-Cu as long as RS+M; 2r-m separated from 2r-rs by 0.37 times its own length, located distal to middle of cell 2mcu; 3r-m separated from apex of cell 3r by half its length, 1.4 times as long as 2r-m; 1m-cu short, 0.45 times and 0.2 times as long as 2-Cu and 3-Cu, respectively; 1cu-a bent distinctly towards wing apex, 0.83 times as long as 2-Cu, distal to middle of cell 1mcu; 1-Cu 1.63 times as long as 2-Cu; 2m-cu oblique towards wing base and straight, distal to middle of cell 3rm, cell 3rm widening towards apex; cell 1mcu 1.48 times as long as wide, 0.73 times as long as cell 2rm; cell 2rm almost as long as 3rm, and 0.66 times as long as and 0.54 times as wide as, cell 2mcu, cell 3r twice as long as 2r.

##### Material examined.

Holotype, No. CNU-HYM-NN-2012148.

##### Distribution.

Jiulongshan Formation; Daohugou Village, Shantou Township, Ningcheng County, Inner Mongolia, China (41°18.979’N, 119°14.318’E); latest Middle Jurassic of the Bathonian-Callovian boundary.

##### Etymology.

The species epithet is derived from the Latin word “elegantulus”, meaning graceful, referring to the habitus of this well preserved specimen.

### Key to the species of *Prolyda* Rasnitsyn, 1968

**Table:** 

1	Forewing with 2r-m and 2r-rs aligned	**2**
–	Forewing with 2r-m distal to 2r-rs	**3**
2	Pterostigma completely sclerotized; 2m-cu located distal to cell 3rm; cell 3rm widening toward wing apex	***Prolyda karatavica***
–	Pterostigma membranous basally and sclerotized apically; 2m-cu located at middle of cell 3rm, and cell 3rm not widened toward apex	***Prolyda dimidia* sp. n.** (Fig. 2A)
3	Forewing with 2m-cu distal to middle of cell 3rm	**4**
–	Forewing with 2m-cu proximal to middle of cell 3rm	***Prolyda depressa***
4	1-RS short, approx. 0.4 times as long as 1-M; RS+M twice as long as 2-M; pterostigma completely sclerotized	***Prolyda elegantula* sp. n.** (Fig. 3E)
–	1-RS long, almost equal to 1-M in length; RS+M equal to 2-M in length; pterostigma partly sclerotized	***Prolyda xyelocera***

## Discussion

*Prolyda* were erected based on two species, *Prolyda karatavica* and *Prolyda xyelocera* (Rasnitsyn 1968). A third species *Prolyda depressa* was described and illustrated by Rasnitsyn (1969). All three species were collected from the early Late Jurassic (Oxfordian or Kimmeridgian) Karatau assemblage of southern Kazakhstan (Kirichkova and Doludenko 1996, Rasnitsyn and Zhang 2004). *Prolyda dimidia* sp. n. and *Prolyda elegantula* sp. n., from the latest Middle Jurassic, are the oldest records of the genus, and dated *Prolyda* further back in time. Furthermore, the wing venation of *Prolyda* in the two epochs is relatively stable, except for only a few characters liable to fluctuate among xyelydids and related taxa, e.g., degree of sclerotization of pterostigma, the relative positions of 2r-m and 2r-rs, and position of vein 2mcu relative to cell 3rm.

*Prolyda* usually possesses a relatively large mesoprescutum, which is almost half the length of the mesonotum, and the mesoscutellum nearly reaches the posterior margin of mesoprescutum. *Prolyda dimidia* sp. n. and *Prolyda elegantula* sp. n. do not have preserved antennae. However, as shown in *Prolyda karatavica* and *Prolyda xyelocera*, *Prolyda* are characterized by an enlarged first flagellomere, which is as long as the head and several times as long as the second flagellomere (Rasnitsyn 1968, 1969). As suggested by Wang et al. (2015a), on basis of the ancestral-state reconstructions of first flagellomere among taxa of Pamphilioidea, that *Prolyda* might have revert to the elongate plesiomorphic state.

## Supplementary Material

XML Treatment for Prolyda

XML Treatment for Prolyda dimidia

XML Treatment for Prolyda elegantula
